# Supplementary material for: A Nomogram Predicting Progression Free Survival in Patients with Gastrointestinal Stromal Tumor Receiving Sunitinib: Incorporating Pre-Treatment and Post-Treatment Parameters
Source: Cancers (Basel). 2021 May 25;13(11):2587. doi: 10.3390/cancers13112587 (PMC8197516; doi:10.3390/cancers13112587)
Supplement: Supplementary file 1 [file cancers-13-02587-s001.zip › cancers-1229025-supplementary.pdf]

Table 1. Summary of pivotal trials with sunitinib use after imatinibfailure.

	Phase I/II (Morgan, 2005)	Phase III (Demitri, 2006)	Phase II (Geoge, 2009)
Eligible patients	After imatinib failure		
Sunitinib dose	50mg (schedule 4/2)		37.5mg (fixed daily dose)
Design	Single arm (N=97)	Sunitinib (N=207) vs. placebo (N=105)	Morning (N=30) vs. Evening dose (N=30)
ORR	7%	7%	-
CBR	54%	58%	53%
PFS	7.8 months	27.3 weeks	34 weeks (around 8.5 months)
OS	19 months	73.9 weeks	107 weeks (around 26 months)

Supplementary Table 2. The adverse events and laboratory abnormalitieswith highest grading detected during the usage of sunitinib in the study

	Any Grade	Grade I-II	Grade III
Any toxicity	100 (91.7%)	65 (59.6%)	35 (32.1%)
Anemia	70 (64.2%)	49 (45.0%)	21 (19.2%)
Hand foot skin reaction	43 (39.4%)	27 (24.7%)	16 (14.7%)
Diarrhea	42 (38.5%)	39 (35.8%)	3 (2.7%)
Fatigue	37 (33.9%)	37 (33.9%)	0 (0%)
Hypertension	28 (25.7%)	24 (22.0%)	4 (3.7%)
Anorexia	26 (23.9%)	26 (23.9%)	0 (0%)
Leukopenia	29 (26.6%)	26 (23.9%)	3 (2.7%)
Thrombocytopenia	27 (24.8%)	23 (21.1%)	4 (3.7%)
Edema	19 (17.4%)	19 (17.4%)	0 (0%)
Hepatic toxicity	15 (13.8%)	10 (9.2%)	5 (4.6%)
Mucositis	13 (11.9%)	13 (11.9%)	0 (0%)
Hypothyroidism	5 (4.6%)	5 (4.6%)	0 (0%)
Myalgia	1 (0.9%)	1 (0.9%)	0 (0%)

Supplementary Table 3. Hazard ratios of predictor variables in multivariate analyses

Predictor variables	Hazard ratio (HR)	95% CI of HR		P value
		Lower	Upper	
Gender				
Male	1			
Female	2.085	1.263	3.440	0.004
ECOG				
0	1			
1/2	1.482	0.802	2.738	0.209
3	3.391	1.519	7.569	0.003
Lymphocyte count				
≦858	0.981	0.562	1.714	0.947
>858	1			
Platelet/lymphocyte ratio				
≦270	1			
>270	1.858	1.119	3.085	0.017
Body mass index (kg/m$^{2)}$				
<18.5	1.964	0.818	4.714	0.131
18.5-27	1			
>27	1.687	0.893	3.186	0.107
Sarcopenia				
Yes	2.310	1.170	4.561	0.016
No	1			
Metastatic site				
Non-liver	1			
Liver	2.195	1.272	3.786	0.005
Sunitinib dose (mg)				
25	3.401	1.164	9.934	0.025
37.5	3.036	1.198	7.699	0.019
50	1			
Hypertension				
No	2.127	1.178	3.840	0.012
Yes	1			
Diarrhea				
No	1.629	0.976	2.720	0.062
Yes	1			
Hand foot syndrome				
No	2.948	1.802	4.824	<0.0001
Yes	1			

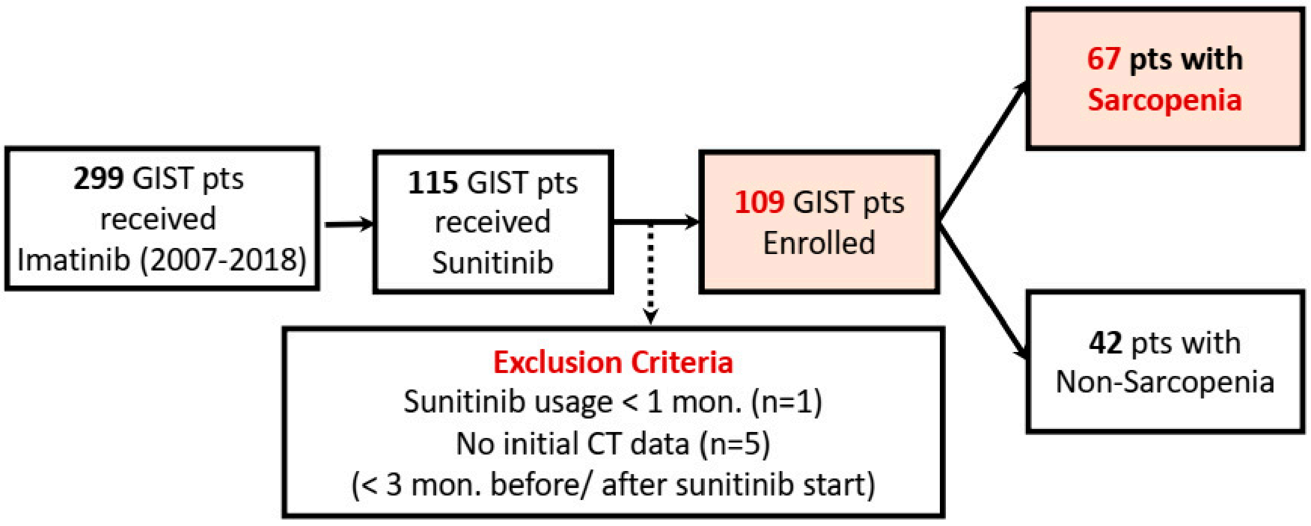

Figure S1： The flow chart of patient selection

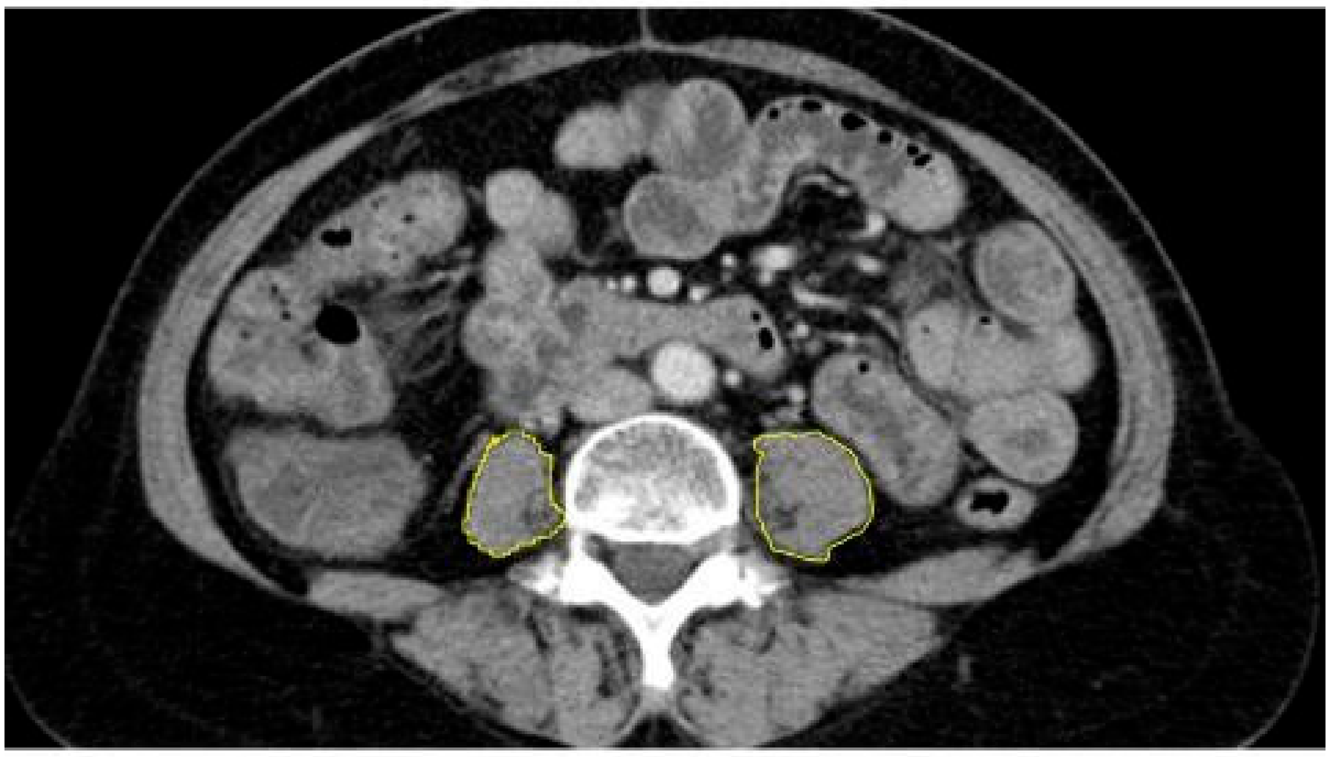

Figure S2: The measurement of total psoas muscle area (TPA)
